# An Exploratory Factor Analysis of Sensor-Based Physical Capability Assessment

**DOI:** 10.3390/s19102227

**Published:** 2019-05-14

**Authors:** Alice Coni, Sabato Mellone, Marco Colpo, Jack M. Guralnik, Kushang V. Patel, Stefania Bandinelli, Lorenzo Chiari

**Affiliations:** 1 Department of Electrical, Electronic and Information Engineering “Guglielmo Marconi” (DEI), University of Bologna, 40136 Bologna, Italy; sabato.mellone@unibo.it (S.M.); lorenzo.chiari@unibo.it (L.C.); 2 Health Sciences and Technologies -Interdepartmental Center for Industrial Research (HST-ICIR), University of Bologna, 40126 Bologna, Italy; 3 Geriatric Unit, Local Health Unit Tuscany Centre, 40125 Firenze, Italy; marco.colpo@hotmail.it (M.C.); stefania1.bandinelli@uslcentro.toscana.it (S.B.); 4 Division of Gerontology, Department of Epidemiology and Public Health, University of Maryland School of Medicine, Baltimore, MD 21201, USA; jguralnik@som.umaryland.edu; 5 Department of Anesthesiology & Pain Medicine, University of Washington, Seattle, WA 98195, USA; kvpatel@uw.edu

**Keywords:** physical capability assessment, instrumented functional test, exploratory factor analysis, older adults

## Abstract

Physical capability (PC) is conventionally evaluated through performance-based clinical assessments. We aimed to transform a battery of sensor-based functional tests into a clinically applicable assessment tool. We used Exploratory Factor Analysis (EFA) to uncover the underlying latent structure within sensor-based measures obtained in a population-based study. Three hundred four community-dwelling older adults (163 females, 80.9 ± 6.4 years), underwent three functional tests (Quiet Stand, QS, 7-meter Walk, 7MW and Chair Stand, CST) wearing a smartphone at the lower back. Instrumented tests provided 73 sensor-based measures, out of which EFA identified a fifteen-factor model. A priori knowledge and the associations with health-related measures supported the functional interpretation and construct validity analysis of the factors, and provided the basis for developing a conceptual model of PC. For example, the “Walking Impairment” domain obtained from the 7MW test was significantly associated with measures of leg muscle power, gait speed, and overall lower extremity function. To the best of our knowledge, this is the first time that a battery of functional tests, instrumented through a smartphone, is used for outlining a sensor-based conceptual model, which could be suitable for assessing PC in older adults and tracking its changes over time.

## 1. Introduction

Physical capability (PC) can be defined as “a person’s ability to do the physical tasks of everyday living” [1]. It is also defined as one of the domains characterizing the healthy aging phenotype recommended for the assessment of older adults by the National Institutes of Health (NIH) [2]. Assessment of the PC was previously achieved using questionnaires and clinical rating scales. Such an assessment is particularly relevant in older adults and is an essential component of comprehensive geriatric assessment in order to develop an overall plan for prevention, treatment, and long-term follow-up. A variety of tools has been proposed in the literature for assessing PC, but several might be sub-optimal for a number of reasons. Some of them suffer from ceiling or floor effect limitations. Some may not be responsive enough to measure slight improvement or deterioration in an older adult’s ability and all self or proxy-reported outcomes may suffer from misreporting. In-lab functional tests are also commonly used, which are quick, simple to administer and make use of inexpensive equipment like a stopwatch or a ruler. Conventional outcome measures of these tests are easily interpretable and therefore widely employed to assess levels of physical function in older adults [3]. Performance on balance tests, gait speed, ability to repeatedly rise from a chair, have been used to characterize PC and predict subsequent health outcomes in community-dwelling populations [1,4,5]. These tests are often administered together, such as in the Short Physical Performance Battery [6] which computes a composite score assessing walking speed, standing balance, and sit-to-stand. This summary score has been demonstrated to have high reliability, validity, and responsiveness [7] and to be predictive of adverse health-related outcomes in community-dwelling older persons [6,8]. Advances in body-worn inertial sensor technology favored the proliferation of instrumented functional tests in which sensor-based measures enrich the outcome of a conventional test. Sensor-based measures are extracted from inertial sensor signals, i.e., accelerometers and gyroscopes, and can provide a more comprehensive assessment of a person’s mobility and balance, well beyond the simple time to perform the test or the distance covered [9].

Instrumented functional tests can qualitatively and quantitatively characterize a wide range of abilities by introducing unique features such as the stepping variability/regularity or the symmetry and coordination of steps during gait [10], the smoothness of postural transitions [11], or the complexity of balance control during postural sway [12].

### State of the Art

Recent literature suggests that a battery of instrumented functional tests, including postural sway assessment, gait analysis, and the assessment of the lower limb strength, may be used to obtain an objective and more detailed picture of a person’s PC. A recent review showed the importance to create consensus in the clinical and research community on a recommended set of functional tests and sensor configurations to standardize the outcome tools and increase comparability between studies [13]. Godfrey et al. showed that the use of standardized instrumented protocols has practical implications in large scale interventions and could also be extended to studies involving pathology, limiting ‘human-error’, and providing the added dimension of novel sensor-based characteristics [9,14]. However, since a single instrumented test produces a large number of sensor-based measures, the clinical interpretation, and validity of these measures, as well as their association with measures obtained from other functional tests, must be carefully analyzed. Covariance among sensor-based measures is high, suggesting redundancy and the need to identify key variables without compromising selectivity. Methods for feature reduction can be found in the literature [15,16,17]. However, these statistical or machine learning methods, address only the issue of reducing the dimension of the dataset. They might provide better results in terms of prediction error, but they select features that are specific for the outcome, without considering the underlying conceptual structure of the initial dataset. A suggested approach to provide a simplified framework is to group the instrumented features into a small number of independent domains using Exploratory Factor Analysis (EFA) [18]. EFA is a multivariate statistical approach widely used in the social, health, biological, and, sometimes, physical sciences, which reduces the number of variables, without imposing a preconceived structure on the outcome. The advantage of such an approach is to reduce the redundancy across variables, by examining the relationships between them [19]. EFA attempts to discover the nature of the latent constructs influencing a set of measured variables [20]. It is based on the common factor model, which proposes that common underlying factors and unique factors influence each observed variable. Unique factors are related to measurement error and variation in the data. Variables that are highly correlated are likely to be influenced by the same factor, while different factors likely influence those that are relatively uncorrelated. Hence, determining the influence of a latent factor on the measured variables, it is possible to indirectly measure the latent construct, which is also commonly referred to as a factor, underlying construct, or unobserved variable. EFA is also used to generate factor scores, representing values of the underlying constructs for use in other analyses. Factor scores are composite variables which provide information about the placement of the person on the factor [21]. Thurstone [22] computed the regression factor scores using a least squares regression approach to predict the location of each individual on the factor. One of the goals of EFA is to provide a conceptual interpretation of the structure, labelling the factors. Each measured variable is assumed to be linearly related to each factor. The corresponding factor loading represents the strength of this relationship, which can be interpreted as a standardized regression coefficient.

The so defined latent factors constitute the conceptual model and may be used to transform datasets containing a high number of correlated sensor-based measures into clinically interpretable information, described in terms of health-related relevant domains. Such an approach has been widely adopted to characterize gait of both community-dwelling older adults and people at risk of falling, or affected by Dementia or Parkinson’s Disease (PD) [23,24,25,26,27,28,29]. These studies developed and validated a conceptual gait model from a set of instrumented temporal gait parameters extracted from a computerized walkway with embedded pressure sensors (GaitRite™). Although the GaitRite™ is a standard tool used to capture gait data [30], wearable inertial sensors provide a valid alternative allowing the extraction of a high number of features in a wide variety of environments and including movements like turning and postural transitions [31]. Recently, two conceptual gait models obtained from body-worn monitors and GaitRite™ data have been compared, and these two models have shown high congruence [32]. However, these conceptual models are based on temporal parameters, and the omission of measures like step/stride regularity, jerk, and RMS acceleration might lead to a loss of useful information. Indeed, as an example, a recent study showed that not all information about impaired PD gait could be captured by measuring spatiotemporal information [33].

Furthermore, these additional measures showed to be related to different health conditions during dynamic and static balance assessment [34,35]. We have found in the literature only a few models describing other functional abilities like static and dynamic balance. Horak et al. developed a model to discover independent domains of balance and gait from instrumented measures extracted through 6 wearable inertial sensors [36]. Our previous works explored and demonstrated the possibility to define an interpretative model for the assessment of PC making use of an EFA on the features derived from the smartphone-based Timed Up and Go test [37,38]. TUG and chair stand test have shown to detect functional status in healthy community-dwelling adults [39]. Furthermore, instrumented chair stand test has shown to be more strongly associated with participant health status, functional status and physical activity [40]. However, we found no studies exploring the possibility to describe domains of this test.

The definition of a sensor-based conceptual model for a comprehensive assessment of the older adults’ PC is the central aim of this work.

## 2. Materials and Methods

### 2.1. Participants

A subsample of 304 community-dwelling older adults (163 females, 80.9 ± 6.4 years old, range 65–98) from the InCHIANTI cohort study (ClinicalTrials.gov NCT01331512) [41] was assessed within the framework of the EU FARSEEING project [42]. Ethical approval was obtained by the Local Ethical Committee (approval number: 584/2012).

### 2.2. Health-Related Measures

Health-related measures included the Mini-Mental State Examination (MMSE), number of medications, Instrumental Activities of Daily Living (IADL), prospective falls (FALL), the number of falls in the last year (FALL history), Center for Epidemiologic Studies Depression Scale (CES-D), Physical Activity (PA), the Short Physical Performance Battery (SPPB), Hand-Grip strength test (HAND), the lower extremity muscle power measured using the Nottingham leg extensor Power Rig (PWR), Trail Making Test A (TMTA), and Gait speed (see Table 1).

### 2.3. Instrumented Tests

Participants performed three functional tests in a fixed order: the assessment of postural sway in Quiet Standing (QS), the 7-meters Walk (7MW) and the 5-times Chair Stand Test (CST). Not all the subjects were able to complete the whole battery of tests: Table 1 reports the demographic and functional profiles of each subgroup undertaking the tests. The tests were instrumented with a smartphone-based system developed within the FARSEEING project [23]. The smartphone (Galaxy SII or Galaxy SIII, Samsung, accelerometer range ±2 g) was worn at the lower back (fifth lumbar vertebra, L5), taken as reference of the body COM [48], by means of an elastic waist belt. A custom Android application was used for recording tri-axial inertial signals (Anteroposterior, AP, Mediolateral, ML, Vertical, V) from the embedded sensors [29]. Acceleration vector magnitude, measured at the lumbar region, is within the range of the accelerometer (±2 g) in the majority of ADLs [30,31]. That would not be true if the sensor were placed in other positions (chest, head, or limbs). In our study, the only activity that could saturate the acceleration signal is the sitting phase in the CST test. However, the effect produced by the impact between the trunk and the chair would be limited to a few samples and would not affect the assessment of the voluntary stand-to-sit movement. Since Android is not a real-time operating system, which means that the physical sensor access depends on other software tasks running in parallel, the sampling rate is usually not constant. We addressed this issue making use of the absolute time reference in nanoseconds associated with each sample [32]. We verified that on the specific mobile and Android version the actual sampling rate was on a narrow size distribution centered on 100 Hz and it was re-sampled offline at exactly 100 Hz before the signal processing.

The time taken to complete the 7MW and CST tests were also recorded with a stopwatch following the usual protocol. Task segmentation and task-specific feature extraction, implemented in Matlab R2017b [49], were based on state-of-the-art methods to characterize postural sway [50], gait [10] and postural transitions [51]. A set of 73 sensor-based measures were computed [52]. Each test was performed as described below. The respective sensor-based measures are shortly summarized here and described in detail in Table 2, Table 3 and Table 4.

*QS:* Subjects stand for 30 seconds with their arms at their side, feet hip-width apart, wearing shoes, with their eyes closed [53]. Twenty-three sensor-based measures are extracted from: i) the acceleration in ML and AP directions, including measures in the time and frequency domains, and ii) the estimated displacement of the body center of mass [50], computed in the time domain to quantify the amount and direction of sway.

*7MW:* Subjects walk 7 meters at a comfortable and safe pace. The start and stop locations are marked on the floor [53]. Gait speed is computed as the distance covered divided for the total time taken to complete the test. Nineteen sensor-based measures are extracted from the acceleration in ML, AP and V direction to describe temporal gait parameters and measures of smoothness, regularity, and coordination [10,54].

*CST:* Subjects start seated on a chair with arms folded across the chest and with their back against the chair’s backrest. On the command “go”, they stand up and sit down five times as quickly as they can [53]. We segmented the CST test into its two sub-phases: Sit-to-Stand and Stand-to-Sit transitions [51]. The AP acceleration and the angular velocity about the ML axis are used to identify postural transitions. Overall, 31 task-specific sensor-based measures are extracted from acceleration and angular velocity in AP, ML and V direction to quantify mean values and standard deviations across repetitions of relevant parameters of the two sub-phases.

### 2.4. Definition of the Conceptual Model

**Figure 1:**
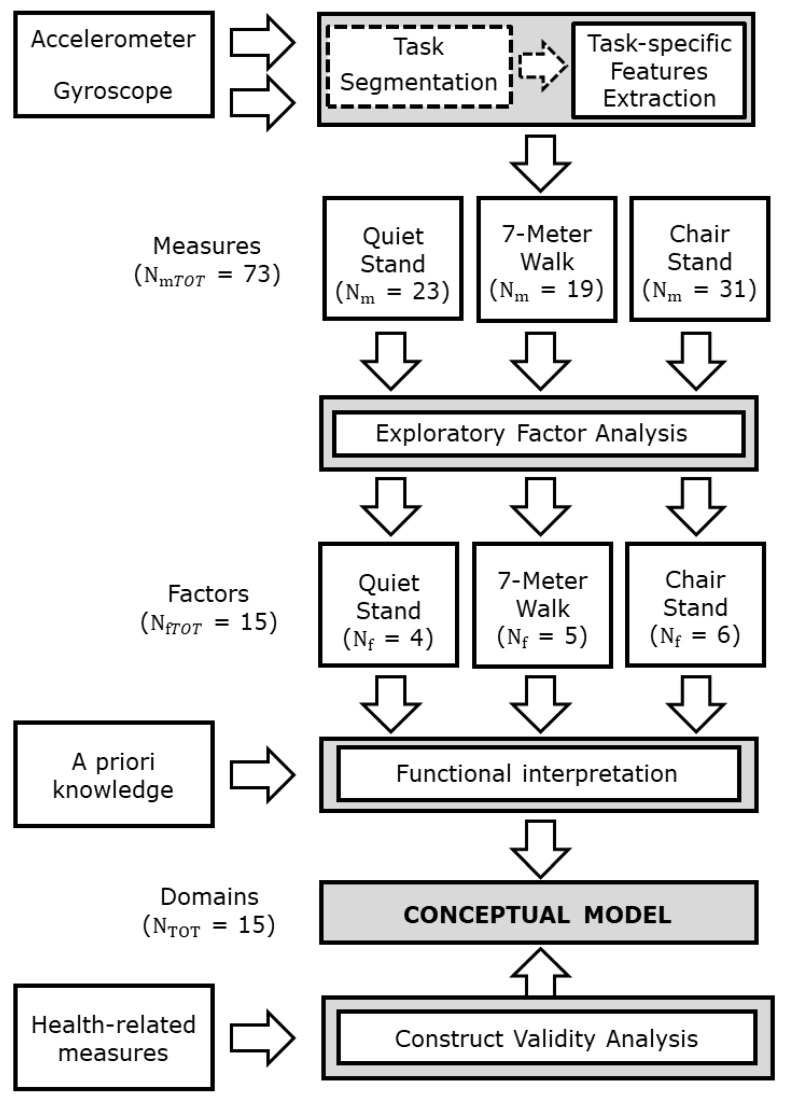
Flowchart of the conceptual model development process. *N_m_* is the number of sensor-based measures extracted from each test, *N_f_* is the number of factors and *N_TOT_* is the total number of domains constituting the conceptual model.

For each instrumented test one EFA was applied to the related sensor-based measures to reduce the dimension of the dataset and to discover the underlying relationships between measures. Since the EFA is based on the assumption of normally distributed data, the jerk scores were log transformed, and all the sensor-based measures were standardized to zero mean and unit variance before EFA. Varimax rotation was used to derive orthogonal factor scores. We considered relevant sensor-based measures with factor loading greater than 0.5 as the absolute value. For each EFA, a scree plot (Parallel analysis) was used to determine the minimum number of factors to retain. To retain as much of the original information as possible, we verified that each resulting factor structure explained at least 70% of the total variance [58]. We hence defined a conceptual model for PC assessment based on the EFA results: We mapped each factor into a specific conceptual domain. Based on our a priori knowledge, the sensor-based measures that contribute to each factor were analyzed and functionally interpreted to identify the corresponding construct. These constructs represent the domains of the conceptual model. Figure 1 shows the flowchart of the conceptual model development process.

### 2.5. Statistical Analysis

Once the constructs (domains) of the conceptual model were defined, we analyzed the following associations: (i) the association between domains in the conceptual model, (ii) the associations between domains and health-related measures, and (iii) the association between health-related measures. The first two associations were performed to investigate the construct validity, while the third analysis was performed to investigate the relationships between health-related measures and whether the covariates influence them. These associations were investigated by computing linear regression analyses. We selected a limited number of covariates from the ones that were available (i.e., age, gender, cognitive status and anthropometric measures) which are known to affect both the heath-related measures and physical performance. Each linear regression analysis was performed two times: first, without adjusting for any covariate and then, adjusting for Age, Gender, Height, Weight, MMSE and number of medications. Finally, the two results were compared to assess the effect of these covariates on the relationships.

We used Bland-Altman analysis to assess the agreement between the smartphone and the stopwatch in measuring the time taken to perform the 7MW and CST tests.

EFA and statistical analyses were performed using RStudio (version R 3.4.3) [59].

## 3. Results

**Figure 2:**
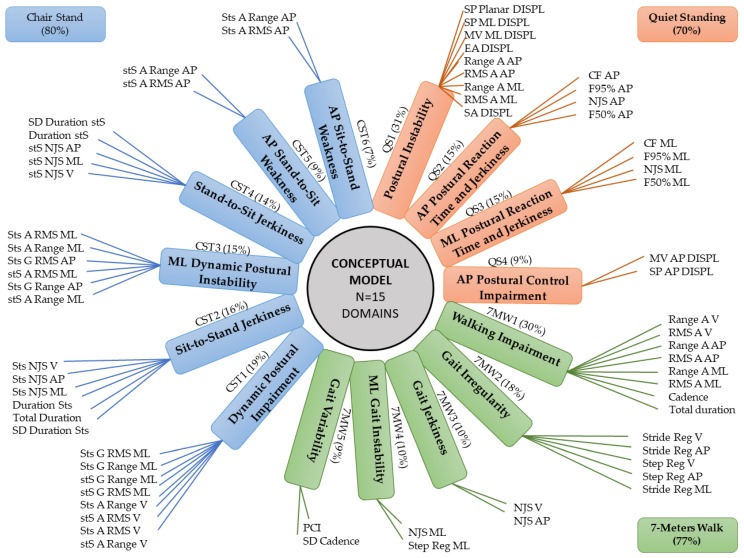
Sensor-based measures contributing to each factor and corresponding domain of the conceptual model for each instrumented test (Orange: Quiet Standing, QS, Green: 7-Meters Walk, 7MW, Blue: Chair Stand, CST). ACRONYMS: A: Accelerometer; AP: Antero-Posterior; CF: Centroidal Frequency; DISPL: displacement; EA: Ellipse Area; FD: Frequency Dispersion; F50%: median frequency, F95%: frequency bandwidth; G: Gyroscope; M: Mean; ML: Medio-Lateral; MV: Mean Velocity; NJS: Normalized Jerk Score; PCI: Phase Coordination Index; Reg: Regularity; RMS: Root Mean Square; SA: Sway Area; SD: Standard Deviation; SE: Spectral Entropy; SP: Sway Path; Sts: Sit to Stand; stS: Stand to Sit; V: Vertical.

Sensor-based measures contributing to each factor obtained from the EFA performed on each test of the battery, percentage of the explained variance and corresponding domains are shown in Figure 2. The a priori knowledge and the functional interpretation of the factors were used for labelling the domains making up the model. As an example, the MV AP DISPL and SP AP DISPL participate to the fourth factor of the QS conceptual model (QS4, see Table 5). These measures have been related to the effectiveness of the postural control system [55], and for this reason, the domain was then labelled as “AP Postural Control Impairment”. Step and stride regularity participate to the second factor of the 7MW model (7MW2, see Table 6), which was labelled “Gait Irregularity” [10] and so forth.

### 3.1. QS Factor Model

The EFA grouped 19 out of 23 sensor-based measures into 4 factors, accounting for 70% of total variance (see Table 5). The resulting independent domains were labeled as: “Postural Instability”, “AP Postural Reaction Time and Jerkiness”, “ML Postural Reaction Time and Jerkiness”, “AP Postural Control Impairment”.

### 3.2. MW Factor Model

The EFA grouped all 19 sensor-based measures into 5 factors, accounting for 77% of total variance (see Table 6). The resulting independent domains were labeled as: “Walking Impairment”, “Gait Irregularity”, “Gait Jerkiness”, “ML Gait Instability”, “Gait Variability”.

### 3.3. CST Factor Model

The EFA grouped 29 out of 31 sensor-based measures into 6 factors, accounting for 80% of total variance (see Table 7). The resulting independent domains were labeled as: “Dynamic Postural Impairment”, “Sit-to-Stand Jerkiness”, “ML Dynamic Postural Instability”, “Stand-to-Sit Jerkiness”, “AP Stand-to-Sit Weakness”, “AP Sit-to-Stand Weakness”.

### 3.4. Construct Validity Analysis

The results of the construct validity analysis are reported in Table 8 and Table 9. The linear regression analysis between the health-related measures provided results reported in Table 10. Beta coefficients with a *p*-value ≤ 0.05 are bolded.

### 3.5. Bland-Altman Analysis

Limits of agreement between the smartphone- and stopwatch-based duration of 7MW and CST were [−0.58, 3.32] s and [−0.13, 5.98] s, respectively.

## 4. Discussion

We aimed to assess whether a battery of instrumented tests is suitable for obtaining a sensor-based conceptual model for the assessment of the older adults’ PC. Our goal was to explore the possible underlying factor structure of the instrumented measures obtained in the tests. For this purpose, we performed one EFA on each set of sensor-based measures obtained from each instrumented test. Machine and statistical learning methods could lead to better results in terms of prediction of the outcome, but these techniques do not take into account the latent structure of the dataset. Therefore, we included all the features in the model as opposed to identifying specific predictors in the original dataset (i.e., the subset of features that achieves the best prediction performance). The domains of the thus obtained conceptual model, which are a linear combination of the original instrumented measures, could also be used as predictors for an outcome, but through EFA they are built independently of a specific outcome. However, these techniques could be evaluated in future studies. An alternative method to EFA, which also reduces the number of features by building linear combinations of the original set of features, is Principal Component Analysis (PCA). Although PCA and EFA sometimes might produce similar results, they are in fact two distinct techniques. The goal of PCA is data reduction, while the goal of EFA is to discover the latent factors that are responsible for a set of measured variables [60]. Indeed, PCA determines linear combinations of the measured variables retaining as much information as possible, without differentiating between common and unique variance. On the contrary, EFA estimates latent constructs that cannot be measured directly (factors). The potential limitations of using EFA are the following: i) it is an exploratory, data-driven procedure, which it is not designed to test hypotheses or theories; ii) the computation of the same set of features is needed for new predictions; iii) if the sample is not representative enough of the general population, it could produce domains that are sample specific. Despite these limitations, we believe that this is the best approach to provide a simplified structure of our original dataset, since the factors, which are obtained independently of a specific outcome, are based on the underlying latent structure. We used the a priori knowledge on the instrumented measures and the functional meaning of the EFA results to approach the conceptual interpretation and naming of the factors. This procedure has led to obtaining the domains constituting the conceptual model finally. Since Varimax rotation was used to derive the factor scores, we can assume that the obtained factors are independent. Indeed, as expected, we found no association between the domains of each functional test (see Table 8). Our work was exploratory, aiming to expand our knowledge and assess the feasibility of developing a model of PC by simplifying the structure from a large number of instrumented measures available. For this purpose, we explored and interpreted the associations between instrumented and standard clinical measures. Table 8, Table 9 and Table 10 show that we found several significant associations in both the unadjusted and adjusted linear regression analyses. The associations that were not explained by the covariates were consistent and confirmed the functional meaning of the domains

In general, the “Walking Impairment” (7MW1) and “Gait Irregularity” (7MW2) domains obtained from the 7MW test were significantly associated with measures of leg muscle power, usual gait speed, and overall lower extremity function. After adjusting for the covariates, two domains were not associated with any health-related measures (see Table 9): the “ML Postural Reaction Time and Jerkiness” (QS3) of the QS factor model, and the “Gait Jerkiness” (7MW3) of the 7MW factor model. This could be due either to non-linear associations between domains and measures or to their association with other health-related measures that were not included in this study. For example, it has been proposed that the capacities in ML direction may be associated with the risk of falls [61] which may not be adequately described by the history of falls. Furthermore, the lack of correlation between quiet standing and fall history could be due to the small number of falls reported by this healthy and fit population. Indeed, only 6% and 5% of the total population experienced at least 2 falls in the previous and following year respectively (see Table 1). In summary, higher-functioning (both physical, SPPB, and cognitive, TMTA) older adults who were more active (PA) and stronger (HAND, PWR) performed better on the instrumented functional tests. A more detailed discussion of these results follows.

### 4.1. Gait Speed

Gait speed was significantly related to domains of the 7MW and CST both in the unadjusted and adjusted model. This is in agreement with other studies in which gait speed was shown to be a good health indicator for older adults [62]. Conversely, the association between this measure and the capacities to maintain the static balance were explained by the covariates. This finding suggests that gait speed may be a useful measure of dynamic balance, but it might not be useful in predicting the abilities to maintain static balance.

### 4.2. SPPB

The covariates explained the association between the Short Physical Performance Battery (SPPB) score and CES-D, TMTA, “AP Postural Control Impairment” (QS4), and “AP Stand-to-Sit Weakness” (CST5). The SPPB score is a measure of the older adults’ functional capacity and includes tests of balance, gait speed, and repeated chair stands. The higher the SPPB score, the better the adults’ performance. As we expected, older adults with high SPPB score, had less IADL, less falls in the last 12 months (FALL-history), they were more active (PA), stronger (HG and PWR), they had less “Postural Instability” (QS1), they showed less difficulties in walking (gait speed, “Walking Impairment”, 7MW1, “Gait Irregularity”, 7MW2, “Gait Variability”, 7MW5) and in performing the CST test (“Sit-to-Stand Jerkiness”, CST2, “Stand-to-Sit Jerkiness”, CST4, “AP Sit-to-Stand Weakness”, CST6).

### 4.3. IADL

The association between IADL and HG, PR, TMTA and the domains of QS (“Postural Instability”, QS1) and CST (“Sit-to-Stand Jerkiness”, CST2, “Stand-to-Sit Jerkiness”, CST4 and “AP Sit-to-Stand Weakness”, CST6) factor model were explained by the covariates. These results show that older adults who had a higher number of instrumental activities in which they required help were also less active and fit, and they had more difficulties while walking (PA, SPPB, gait speed, “Walking Impairment”, 7MW1, “Gait Irregularity”, 7MW2).

### 4.4. FALL-History

The associations between the number of falls experienced during the last 12 months (FALL-history) and SPPB, “Gait Irregularity” (7MW2) and “Sit-to-Stand Jerkiness” (CST2) were not explained by the covariates. This implies that older adults who experienced more falls showed poorer performances in the domains that require strength. Indeed, they were less fit and less smooth during postural transitions. Gait speed was not related to the history of falls, but older adults who fell more showed a less regular gait. This finding is in agreement with a recent study, in which senior athletes with a history of falling demonstrate poorer performance than those who report no falls, and CST was highly related to fall history, suggesting the need for strength besides the balance in an individual’s ability to prevent falls [63].

### 4.5. CES-D

CES-D is a screening test for depression and depressive disorders. The associations between CES-D and all the health-related measures, the domains of the 7MW (“Walking Impairment”, 7MW1 and “Gait Jerkiness”, 7MW3) and CST (“ML Dynamic Postural Instability”, CST3) factor models, were explained by the covariates. In summary, after adjusting for the covariates, older adults who reported depressive symptoms were less reactive and smooth during the QS test (“AP Postural Reaction Time and Jerkiness”, QS2), and they showed more “Gait Irregularity” (7MW2) and “AP Sit-to-Stand Weakness” (CST6). Since postural transitions need high Range of Motion, older adults with depressive symptoms appear to be less strong and reactive. This result is in agreement with the study by Penninx et al. [64] in which depressive symptoms were predictive for decline in physical performance.

### 4.6. PA

The associations between the declared physical activity (PA) and the CES-D, HG, PR, TMTA, “Gait Jerkiness” (7MW3) and “AP Sit-to-Stand Weakness” (CST6) were explained by the covariates. As expected, older adults who were less active, had a higher number of instrumental activities in which they required help (IADL), they were less fit (SPPB), and less able to walk (gait speed, “Walking Impairment”, 7MW1, “Gait Irregularity” 7MW2).

### 4.7. HAND

The association between Hand-Grip strength test (HAND) and IADL, CES-D, PA, TMTA, “AP Postural Instability” (QS4) and some domains of the 7MW (“Walking Impairment”, 7MW1, “Gait Jerkiness”, 7MW3) and CST (“ML Dynamic Postural Instability”, CST3, “Stand-to-Sit Jerkiness”, CST4, “AP Sit-to-Stand Weakness”, CST6) factor models were explained by the covariates. The performances in the CST test reflect the strength of the lower limbs. Surprisingly, after adjusting for the covariates, no significant associations between upper limbs strength (HG) and CST factor model were found. Older adults with higher HAND were more fit and strong (SPPB and PWR), they had better capacities in maintaining static balance (“Postural Instability”, QS1), and walking (gait speed, “Gait Irregularity”, 7MW2 and “ML Gait Instability”, 7MW4). These results are consistent with the findings of a previous study that highlighted the association between grip-strength and future outcome in aging adults [65].

### 4.8. PWR

After adjusting for the covariates, only the SPPB score, the HAND and the gait speed were significantly associated with the lower limbs strength (PWR). No significant associations between PWR and domains of the QS factor model were found. In our findings, the associations between strength (both HAND and PWR) and the ability to maintain static balance (“AP Postural Control Impairment”, QS4) were explained by the covariates. Older adults who had higher lower limbs strength, showed less difficulties to walk (gait speed, “Walking Impairment”, 7MW1, “Gait Irregularity”, 7MW2) and, as expected, performed better in the CST test (“Sit-to-Stand Jerkiness”, CST2, “ML Dynamic Postural Instability”, CST3, “AP Stand-to-Sit Weakness”, CST5, “AP Sit-to-Stand Weakness”, CST6).

### 4.9. TMTA

The Trail Making Test part A (TMTA) assesses psychomotor speed. Attention and executive function are related to the cognitive control of gait, posture, and balance (6,7). Performance on the TMTA is a strong, independent predictor of mobility impairment, accelerated decline in lower extremity function, and mortality in older community-living adults (8). After adjusting for the covariates, only the association between the TMTA and “AP Postural Control Impairment” (QS4), gait speed, “Walking Impairment” (7MW1) and “Stand-to-Sit Jerkiness” (CST4) were significant.

All these results suggest that the sensor-based model is consistent with the conventional clinical measures of PC. The gait speed and SPPB served as a standard clinical outcome measure of older adults’ PC. The coherence of the information obtained from the instrumented measures and the standard clinical outcome (and other health-related measures) was investigated through the linear regression analysis. As expected, the SPPB score correlates with all the health-related measures (Table 10) whereas this measure doesn’t correlate with all the domains of the conceptual model (Table 9). The domains that were not associated with the health-related measures suggested that they refer to abilities that were not possible to objectively measure with the standard outcome. The interpretation of the construct validity analysis results confirmed that inertial sensors embedded in smartphones can detect and assess the status of different functional domains, adding useful information to the conventional clinical assessment. To the best of our knowledge, this is the first time that conceptual models are used to transform datasets obtained from an instrumented battery of sensor-based functional tests into clinically interpretable information. Such a model can contribute to facilitate the adoption of the sensor-based assessment in everyday clinical practice. However, further validation studies also involving different target groups are needed to deeply investigate such interpretative models.

### 4.10. Case Studies

**Figure 3:**
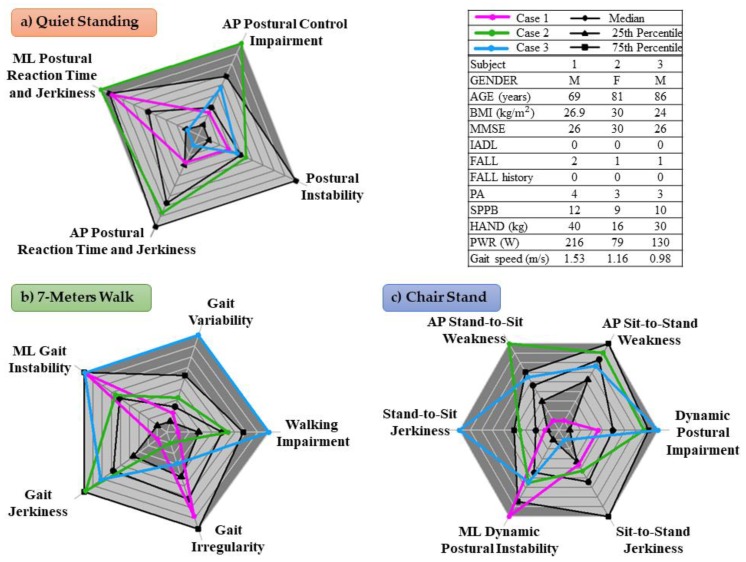
Three radar plots reporting the Quiet Stand (**a**), 7-meters Walk (**b**) and 5-times Chair Stand (**c**) domains of three different case studies. The black lines represent the median value, the 25th and 75th percentiles of the older adults’ factor scores. The dark gray area represents extreme values (very high, above the 75th percentile, or very low, below the 25th percentile). Favorable values of the scores, below the 75th percentile, reflected good performances in the domain.

In this section, a possible scenario in which a clinician could benefit from the additional information provided by the sensor-based conceptual model is presented. Figure 3 shows three radar plots for three different case studies, providing a graphical representation of the conceptual model. The black lines represent the median value, the 25th and 75th percentiles of the scores given to the older adults. The dark gray area represents extreme values (very high, above the 75th percentile, or very low, below the 25th percentile). Favorable values of the scores, below the 75th percentile, reflect good performances in the domain.

Case 1: Based on the clinical assessment of the subject (male, 69 years old) was not at risk of a fall, but 2 prospective falls occurred. As shown in Figure 3, he showed high instability in ML direction during the QS, 7MW and postural transitions (QS3, 7MW4 and CST3 were above the 75th percentile). This may corroborate the idea that ML stability is crucial to prevent falls in community-dwelling older adults [66,67].

Case 2: The older adult (female, 81 years old) had all the health-related measures within their reference values, but she had poor strength (low HAND and PWR). The weakness is reflected in poor ability to maintain the static balance: High “ML Postural Reaction Time and Jerkiness” (QS3) and “AP Postural Control Impairment” (QS4), confirming the findings reported elsewhere [68]. She showed also high “Gait Jerkiness” (7MW3) and poor ability to perform the CST test: high “Dynamic Postural Impairment” (CST1), “Stand-to-Sit Jerkiness” (CST4) and “AP Stand-to-Sit Weakness” (CST5).

Case 3: The older adult (male, 86 years old) had all the health-related measures within their reference values, except for the gait speed, which was below 1 m/s. This cut-off point has been related to the risk of adverse health outcomes and disabilities [5,69]. Indeed, the Radar Plots show that his capacities to maintain static balance are not compromised, but he had difficulties while walking (high “Walking Impairment”, 7MW1, “ML Gait Instability”, 7MW4, and “Gait Variability”, 7MW5) and while performing postural transitions (high “Dynamic Postural Impairment”, CST1 and “Stand-to-Sit Jerkiness”, CST4, and “AP Stand-to-Sit Weakness”, CST5).

## 5. Conclusions

To the best of our knowledge, this is the first time that a battery of functional tests, instrumented through a smartphone, is used for outlining a sensor-based conceptual model (Figure 2), which is suitable for physical capability assessment of older adults. EFA allowed us to reduce the number of sensor-based measures taken from instrumented functional tests and find domains with clear functional meaning. The interpretation of the significant associations suggests that such domains confirm and expand information obtained with clinical testing and provide quantitative information about several mobility skills that are usually not captured by conventional outcomes. This exploratory research shows that instrumented functional testing has the potential to advance the quality of current mobility assessments; enhance our understanding of an individual’s true physical capabilities; and disclose subtle changes in physical capabilities that would otherwise remain undetected. Increasing our understanding and the sensitivity of mobility assessment is of the utmost importance since it may enable earlier detection of functional decline and identify therapeutic targets for rehabilitation. Further work is needed to evaluate whether this more detailed information adds to our ability to predict adverse outcomes, over and above clinical testing like gait speed and SPPB.

## Figures and Tables

**Table 1:** Demographic and functional profiles of each subgroup undertaking the three functional tests.

	Total Population	QS	7MW	CST
Sample Size	304	204	201	173
Gender (females)	163 (54%)	97 (48%)	95 (47%)	80 (46%)
AGE, years	80.90 (6.37)	79.46 (6.43)	79.39 (6.44)	79.35 (6.25)
Weight, kg	69.60 (13.30)	70.52 (13.13)	70.35 (13.18)	70.90 (13)
Height, cm	159.72 (9.53)	160.51 (9.21)	160.50 (9.20)	160.79 (8.88)
MMSE, (range 0–30)	27.25 (1.77)	27.41 (1.76)	27.41 (1.77)	27.53 (1.72)
Medications >=4	169 (56%)	98 (48%)	95 (47%)	83 (48%)
IADL >= 1, (range 0–8)	114 (38%)	49 (24%)	47 (23%)	39 (23%)
FALL >=2	16 (5%)	5 (2%)	5 (2%)	3 (2%)
FALL history >=2	19 (6%)	11 (5%)	10 (5%)	7 (4%)
CES-D >= 16, (range 0–60)	106 (35%)	58 (28%)	57 (28%)	49 (28%)
PA, categories, (range 1–7)	2.91 (1.01)	3.16 (0.98)	3.15 (0.99)	3.24 (1.01)
SPPB, (range 0–12)	8.72 (3.18)	9.80 (1.98)	9.82 (1.98)	9.92 (1.87)
HAND, kg	26.98 (9.26)	28.85 (8.98)	28.81 (9.00)	29.09 (8.97)
PWR, watt	88.69 (51.28)	94.71 (51.28)	95.21 (51.62)	94.96 (48.54)
TMTA, s	78.37 (43.94)	70.51 (36.50)	70.69 (36.59)	69.48 (35.19)
Gait speed, m/s	1.11 (0.26)	1.15 (0.25)	1.15 (0.25)	1.15 (0.24)
Values are presented as mean (SD) or number (%) unless otherwise indicated				

ACRONYMS: MMSE: Mini-Mental State Examination; IADL: Instrumental Activities of Daily Living, i.e., the number of instrumental activities in which the person requires help (e.g., preparing meals, performing housework, getting to places outside of walking distance, managing medications, etc.) [43]; FALL: Prospective falls, number of falls occurred in the following year; FALL history: The number of falls in the last year declared during the assessment; CES-D: Center for Epidemiologic Studies Depression Scale, a questionnaire used to assess depressive symptoms (32); PA: Physical Activity, assessed through a questionnaire [44]; SPPB: Short Physical Performance Battery, a measure of mobility function [8]; HAND: The Hand-Grip strength test [45] kg, stronger hand; PWR: The lower extremity muscle power measured using the Nottingham leg extensor Power Rig [46], watt; TMTA: Trail Making Test A, a neuropsychological test that assesses various cognitive abilities, including visual-conceptual, visuospatial, and visual-motor tracking [47], s; Gait speed: obtained from the distance covered (7 meters) and the total time taken to complete the test, m/s.

**Table 2:** Sensor-based features extracted from the QS test.

Feature	Sensor	Description
CF AP ML [55,56]	Accelerometer	Centroidal frequency; frequency at which spectral mass is concentrated. Spectral moments are needed for the estimate: $\mu_{0}=\overset{N}{\underset{i=1}{\sum }}PSD_{i}=TP;\;\mu_{2}=\overset{N}{\underset{i=1}{\sum }}\;f_{i}^{2}\;PSD_{i};\;CF=\sqrt{\frac{\mu_{2}}{\mu_{0}}}$ where *PSD* is the Power Spectral Density of the signal, *f* is the frequency vector, and *N* is the total number of points of the *PSD*. Frequencies below 0.15 Hz are usually ignored.
EA DISPL [55,56]	Accelerometer, Displacement	The 95% confidence Ellipse Area is the area of the confidence ellipse enclosing 95% of the points on the sway trajectory. The accelerometer-based postural parameter can be defined by analogy with the parameter based on the displacement.
F_50%_ AP ML [55,56]	Accelerometer	Median frequency; frequency below which 50% of total signal power (*TP*) is present. Starting from the Power Spectral Density (*PSD*) of the signal: $g_{n}=\overset{n}{\underset{i=1}{\sum }}PSD_{i}\;;\;F_{50\%}=f_{n}\;,\min_{n}:g_{n}\;\geq 50\%TP\;$ where the second formula means that *F*_50%_ is the frequency, *f*, corresponding to the nth index which is the smallest index such that g(n) is ≥50% of the total power. The total power is equal to g(N) where *N* is the total number of points of the *PSD*. Frequencies below 0.15 Hz are usually ignored.
F_95%_ AP ML [55,56]	Accelerometer	Frequency below which 95% of total signal power (*TP*) is present. Starting from the Power Spectral Density (*PSD*) of the signal: $g_{n}=\overset{n}{\underset{i=1}{\sum }}PSD_{i}\;;\;F_{95\%}=f_{n}\;,\min_{n}:g_{n}\;\geq 95\%TP\;$ where the second formula mean that *F*_95%_ is the frequency, *f*, corresponding to the nth index which is the smallest index such that g(n) is ≥95% of the total power. The total power is equal to g(N) where *N* is the total number of points of the *PSD*. Frequencies below 0.15 Hz are usually ignored.
FD AP ML [55,56]	Accelerometer	Frequency dispersion; unitless measure of the variability of the power spectral density frequency content (zero for pure sinusoid; increases with spectral bandwidth to one). Spectral moments are needed for the estimate: $\mu_{0}=\overset{N}{\underset{i=1}{\sum }}PSD_{i}=TP;\;\mu_{1}=\overset{N}{\underset{i=1}{\sum }}f_{i}\;PSD_{i}\;;\;\mu_{2}=\overset{N}{\underset{i=1}{\sum }}f_{i}^{2}\;PSD_{i};\;FD=\sqrt{\frac{1-\mu_{1}^{2}}{\mu_{0}\mu_{2}}}\;\;$ where *PSD* is the Power Spectral Density of the signal, *f* is the frequency vector, and *N* is the total number of points of the *PSD*. Frequencies below 0.15 Hz are usually ignored.
MV DISPL AP ML [55,56]	Accelerometer, Displacement	Mean Velocity of the postural sway computed as the median of the absolute value of the time series obtained integrating the acceleration: $MV=median\left(\int_{Tstart}^{Tend}a\left(t\right)dt\right)$ where a is the acceleration component m/s^2^, *Tend/Tstart* are the end and the beginning of the observation time respectively. An alternative definition can be based upon the Sway Path (SP) of the displacement: $MV=\left(\frac{SP}{T_{end}-T_{start}}\right)$
NJS AP ML [35,54]	Accelerometer	Normalized Jerk Score of the acceleration: $NJS=\sqrt{\frac{T^{5}}{2SP^{2}}}\int_{T_{start}}^{T_{end}}{\dot{\left(a\right)}}^{2}dt$ where *T* is the duration (*T_end_* − *T_start_*) of the considered component, *a* is the acceleration measured in m/s^2^, and *SP* is the Sway Path
Range AP ML	Accelerometer	Range of the signal
RMS AP ML	Accelerometer	Root Mean Square (*RMS*) of the signal, *s* (it is a measure of dispersion): $RMS=\sqrt{\frac{1}{N}}\overset{N}{\underset{i=1}{\sum }}{\left(s_{i}\;-m\right)}^{2}$ where *N* is the total number of points of the signal *s*, and m is the mean value mean(s)
SA DISPL [55,56]	Displacement	Sway Area (*SA*) estimated as the sum of the triangles formed by two consecutive points on the sway trajectory on the horizontal plane (*s_AP_* and *s_ML_)* and the mean point (*m_AP_ and m_ML_)* on the plane: $SA=\frac{1}{2}\overset{N-1}{\underset{i=1}{\sum }}\left|\left(s_{AP,i+1\;}-m_{AP}\right)\left(s_{ML,\;i}-m_{ML}\right)-\left(s_{AP,i}-m_{AP}\right)\left(s_{ML,\;i+1}-m_{ML}\right)\right|$ where *s* is a generic signal, *s_AP_* and *s_ML_* are the two sway components on the horizontal plane. *N* is the total number of points of the signal time series. The accelerometer-based postural parameter can be defined by analogy with the parameter based on the displacement.
SE AP ML [55,56]	Accelerometer	Spectral Entropy Power spectrum entropy of acceleration (unitless).
SP AP ML DISPL SP Planar DISPL [55,56]	Accelerometer, Displacement	Sway Path, the total length of the sway trajectory, computed as the sum of the distances between consecutive points in the time series. When considering a single direction of the sway: $P=\overset{N-1}{\underset{i=1}{\sum }}\left(s_{i+1}-s_{i}\right)$ When considering the sway path on the horizontal plane: $SP=\frac{1}{2}\overset{N-1}{\underset{i=1}{\sum }}{\left(s_{AP,\;i+1}-s_{AP,\;i}\right)}^{2}+{\left(s_{ML,i+1}-s_{ML,\;i}\right)}^{2}$ where *s* is a generic signal, *s_AP_* and *s_ML_* are the two sway components on the horizontal plane. *N* is the total number of points of the signal time series. The accelerometer-based postural parameter can be defined by analogy with the parameter based on the displacement

ACRONYMS: AP: Antero-Posterior; CF: Centroidal Frequency; EA: Ellipse Area; F_50%_: Median Frequency; F_95%_: Frequency below 95% of total signal power; FD: Frequency Dispersion; ML: Medio-Lateral; MV: Mean Velocity; NJS: Normalized Jerk Score; RMS: Root Mean Square; SA: Sway Area; SE: Spectral Entropy; SP: Sway Path; V: Vertical.

**Table 3:** Sensor-based features extracted from the 7MW test.

Feature	Sensor	Description
Duration [s]	Accelerometer/Gyroscope	Total duration of the test
Cadence [steps/min]	Accelerometer	Cadence in the phase of the gait
SD Cadence	Accelerometer	Standard deviation of the Cadence
NJS AP ML V [m] [35,54]	Accelerometer	The Normalized Jerk Score during gait is computed for each step (i.e., between two consecutive heel strikes), then normalized to the step duration, and then averaged across all steps $NJS=\sqrt{\frac{T^{5}}{2}\int_{Tstart}^{Tend}{\left(\dot{a}\right)}^{2}dt}$ where *T* is the duration (*T_end_ − T_start_*), *a* is the acceleration measured in m/s^2^.
PCI [35,57] [-]	Accelerometer	Phase Coordination Index (PCI). PCI measures gait coordination (i.e., the accuracy and consistency of the phase generation). $PCI=PhaseCV+100\cdot \frac{\frac{1}{N}\sum_{i=1}^{N}\left|\varphi_{i}-180^{{}^{\circ}}\right|}{180^{{}^{\circ}}}$ where *PhaseCV* is the Coefficient of Variation of the Phase. *φ_i_* is the ith phase, which measures the step time with respect to the stride time assigning 360° to each stride (gait cycle): $\varphi_{i}=360^{{}^{\circ}}\frac{hs_{S,i}-\;hs_{L,i}}{hs_{L,i+1}-\;hs_{L,i}}$ where *hs_L(i)_* and *hs_S(i)_* denote the time of the ith heel strike of the legs with the long and short step times, respectively.
Range AP ML V [m/s^2^]	Accelerometer	Range of the signal
RMS AP ML V [m/s^2^]	Accelerometer	Root Mean Square (*RMS*) of the signal, *s* (it is a measure of dispersion): $RMS=\sqrt{\frac{1}{N}}\overset{N}{\underset{i=1}{\sum }}{\left(s_{i}\;-m\right)}^{2}$ where *N* is the total number of points of the signal *s*, and m is the mean value mean(s)
Reg [10] AP ML V [10] [-]	Accelerometer	Step and Stride regularity measured by means of the unbiased estimate of the autocorrelation function of the signal *s*: $A_{unbiased}=\frac{1}{N-\left|n\right|}\overset{N-\left|n\right|}{\underset{i=1}{\sum }}s_{i}s_{i+n}$ where *N* is the total number of points of the signal and *n* is the phase shift in number of samples. First dominant period (*A_d1_*) of the autocorrelation coefficient is an expression of the step regularity. Second dominant period (*A_d2_*) of the autocorrelation coefficient is an expression of the Stride regularity

ACRONYMS: AP: Antero-Posterior; ML: Medio-Lateral; NJS: Normalized Jerk Score; PCI: Phase Coordination Index; Reg: Regularity; RMS: Root Mean Square; SD: Standard Deviation; V: Vertical.

**Table 4:** Sensor-based features extracted from the CST Test.

Feature	Sensor	Task	Description
SD Duration	Accelerometer/Gyroscope	Sit-to-Stand, Stand-to-Sit	Standard deviation of the duration of each subtask of the test.
Duration [s]	Accelerometer/Gyroscope	Total, Sit-to-Stand, Stand-to-Sit	Duration of each subtask of the test.
NJS AP ML V [m]	Accelerometer	Sit-to-Stand, Stand-to-Sit	Normalized Jerk Score of the acceleration (it is related with the smoothness of the movement): $NJS=\sqrt{\frac{T^{5}}{2}\int_{Tstart}^{Tend}{\left(\dot{a}\right)}^{2}dt}$ where *T* is the duration (*Tend-Tstart*) of the considered sub-task and *a* is the acceleration measured in m/s^2^.
Range AP ML V [m/s^2^], [°/s]	Accelerometer, Gyroscope	Sit-to-Stand, Stand-to-Sit	Range of the signal, during the considered sub-task of the test
RMS AP, ML, V [m/s^2^], [°/s]	Accelerometer, Gyroscope	Sit-to-Stand, Stand-to-Sit	Root Mean Square (*RMS*) of the signal, *s*, during the considered sub-task of the test (it is a measure of dispersion): $RMS=\sqrt{\frac{1}{N}}\overset{N}{\underset{i=1}{\sum }}{\left(s_{i}\;-m\right)}^{2}$ where *N* is the total number of points of the signal *s*, and m is the mean value mean(s)

ACRONYMS: AP: Antero-Posterior; ML: Medio-Lateral; NJS: Normalized Jerk Score; RMS: Root Mean Square; SD: Standard Deviation; V: Vertical.

**Table 5:** QS factor model: factor loadings of the sensor-based measures, corresponding domains and Cumulative Variance (CV) (Varimax Rotation).

Domain	Postural Instability	AP Postural Reaction Time and Jerkiness	ML Postural Reaction Time and Jerkiness	AP Postural Control Impairment
Factor	QS1	QS2	QS3	QS4
Range A ML	**0.948**	−0.107	−0.145	0.066
RMS A ML	**0.933**	−0.012	−0.250	0.015
SA DISPL	**0.928**	0.011	−0.069	0.129
SP ML DISPL	**0.872**	−0.142	−0.096	0.183
MV ML DISPL	**0.795**	−0.119	−0.105	0.203
EA DISPL	**0.774**	0.056	−0.115	−0.029
SP Planar DISPL	**0.764**	−0.056	−0.082	0.625
Range A AP	**0.706**	0.185	−0.107	0.432
RMS A AP	**0.675**	0.288	−0.144	0.296
CF AP	−0.126	**−0.983**	0.113	−0.003
F95 AP	−0.103	**−0.918**	0.141	−0.068
NJS AP	0.121	**−0.789**	0.106	−0.130
F50 AP	−0.129	**−0.774**	0.102	0.165
CF ML	−0.195	−0.201	**0.957**	−0.024
F_95%_ ML	−0.263	−0.153	**0.872**	−0.048
NJS ML	−0.051	−0.168	**0.849**	−0.109
F_50%_ ML	−0.088	−0.207	**0.794**	−0.010
MV AP DISPL	0.533	−0.006	−0.097	**0.802**
SP AP DISPL	0.626	−0.004	−0.084	**0.765**
SE ML	−0.169	0.03	0.230	−0.041
SE AP	0.117	−0.312	0.094	−0.073
FD ML	−0.066	0.178	−0.156	−0.112
FD AP	0.016	−0.280	0.032	−0.298
CV%	31	46	61	70
Relevant factor loadings (absolute value > 0.5) are bolded				

ACRONYMS: A: Accelerometer; AP: Antero-Posterior; CF: Centroidal Frequency; CV: Cumulative Variance; EA: Ellipse Area; F_50%_: Median Frequency; F_95%_: Frequency below 95% of total signal power; FD: Frequency Dispersion; Medio-Lateral; MV: Mean Velocity; NJS: Normalized Jerk Score; RMS: Root Mean Square; SA: Sway Area; SE: Spectral Entropy; SP: Sway Path; V: Vertical.

**Table 6:** 7MWT factor model: Factor loadings of the sensor-based measures, corresponding domains and Cumulative Variance (CV) (Varimax Rotation).

Domains	Walking Impairment	Gait Irregularity	Gait Jerkiness	ML Gait Instability	Gait Variability
Factors	7MW1	7MW2	7MW3	7MW4	7MW5
Range A V	**−0.916**	−0.030	−0.116	0.035	−0.050
RMS A V	**−0.909**	−0.279	−0.106	0.046	−0.109
Range A AP	**−0.882**	−0.046	−0.157	0.113	−0.102
RMS A AP	**−0.866**	−0.183	−0.119	0.181	−0.140
Range A ML	**−0.735**	0.227	−0.113	0.521	−0.008
RMS A ML	**−0.727**	0.070	0.044	0.678	−0.012
Cadence	**0.708**	0.253	−0.654	0.005	0.047
Total duration	**0.663**	0.394	0.017	0.014	0.237
Stride Reg V	−0.163	**−0.844**	−0.007	−0.120	−0.158
Stride Reg AP	0.087	**−0.823**	−0.008	0.130	−0.174
Step Reg V	−0.279	**−0.737**	−0.001	−0.145	−0.223
Step Reg AP	−0.151	**−0.658**	−0.063	0.072	−0.215
Stride Reg ML	−0.026	**−0.633**	0.176	0.445	0.013
NJS V	−0.125	0.050	**−0.826**	−0.007	0.147
NJS AP	−0.404	−0.176	**−0.662**	0.078	−0.116
NJS ML	−0.179	0.064	−0.389	**0.669**	−0.012
Step Reg ML	−0.065	−0.387	0.204	**0.557**	−0.003
PCI	0.120	0.269	−0.007	0.001	**0.893**
SD Cadence	0.234	0.390	−0.123	−0.017	**0.836**
CV%	30	48	58	68	77
Relevant factor loadings (absolute value > 0.5) are bolded					

ACRONYMS: A: Accelerometer; AP: Antero-Posterior; CV: Cumulative Variance; ML: Medio-Lateral; NJS: Normalized Jerk Score; PCI: Phase Coordination Index; Reg: Regularity; RMS: Root Mean Square; SD: Standard Deviation; V: Vertical.

**Table 7:** CST factor model: Factor loadings of the sensor-based measures, corresponding domains and Cumulative Variance (CV) (Varimax Rotation).

Domains	Dynamic Postural Impairment	Sit-to-Stand Jerkiness	ML Dynamic Postural Instability	Stand-to-Sit Jerkiness	AP Stand-to-Sit Weakness	AP Sit-to-Stand Weakness
Factors	CST1	CST2	CST3	CST4	CST5	CST6
Sts G RMS ML	**−0.904**	−0.008	−0.115	−0.137	−0.003	−0.060
Sts G Range ML	**−0.846**	0.031	−0.16	−0.045	−0.032	−0.292
stS A RMS V	**−0.836**	0.118	−0.195	0.259	−0.103	0.101
stS G RMS ML	**−0.832**	−0.203	−0.142	−0.153	−0.245	−0.042
Sts A Range V	**−0.826**	0.131	−0.213	0.392	0.004	0.071
Sts A RMS V	**−0.809**	0.128	−0.176	0.393	0.086	0.148
stS A Range V	**−0.702**	0.090	−0.23	0.112	−0.395	−0.087
stS G Range ML	**−0.659**	−0.128	−0.173	−0.094	−0.454	−0.151
Sts JS V	−0.207	**0.909**	0.084	0.208	0.118	0.084
Sts JS AP	−0.054	**0.909**	0.071	0.238	−0.102	0.133
Sts JS ML	−0.047	**0.897**	−0.032	0.205	0.15	0.127
Duration Sts	0.027	**0.85**	0.179	0.286	0.331	0.145
SD Duration Sts	0.154	**0.718**	0.024	−0.109	0.026	−0.081
Total Duration	−0.039	**0.706**	0.173	0.529	0.277	0.243
Sts A RMS ML	−0.055	0.019	**−0.938**	−0.048	−0.131	−0.161
Sts A Range ML	−0.118	0.008	**−0.918**	0.013	−0.129	−0.245
Sts G RMS AP	−0.255	−0.086	**−0.651**	−0.012	−0.049	−0.100
stS A RMS ML	−0.126	−0.140	**−0.644**	−0.111	−0.402	−0.049
Sts G Range AP	−0.394	−0.085	**−0.638**	0.084	−0.037	−0.106
stS A Range ML	−0.116	−0.056	**−0.511**	−0.075	−0.504	−0.110
stS JS AP	−0.162	0.226	0.043	**0.905**	0.098	−0.122
stS JS ML	−0.106	0.291	−0.234	**0.859**	0.123	0.218
stS JS V	−0.348	0.304	0.059	**0.836**	0.143	0.173
Duration stS	−0.097	0.404	0.161	**0.778**	0.197	0.338
SD Duration stS	0.210	−0.080	0.151	**0.595**	−0.125	0.090
stS A Range AP	−0.138	−0.164	−0.292	−0.070	**−0.859**	−0.193
stS A RMS AP	−0.208	−0.304	−0.295	−0.136	**−0.764**	−0.284
Sts A Range AP	−0.084	−0.216	−0.304	−0.239	−0.229	**−0.842**
Sts A RMS AP	−0.138	−0.215	−0.315	−0.276	−0.224	**−0.786**
stS G Range AP	−0.320	−0.055	−0.402	−0.056	−0.342	0.068
stS G RMS AP	−0.221	−0.134	−0.449	−0.110	−0.317	0.105
CV%	19	35	50	64	73	80
Relevant factor loadings (absolute value > 0.5) are bolded						

ACRONYMS: A: Accelerometer; AP: Antero-Posterior; CV: Cumulative Variance; G: Gyroscope; ML: Medio-Lateral; NJS: Normalized Jerk Score; RMS: Root Mean Square; SD: Standard Deviation; Sts: Sit to Stand; stS: Stand to Sit; V: Vertical.

**Table 8:** Construct validity analysis: Linear regression between domains.

	*QS1*	*QS2*	*QS3*	*QS4*	*7MW1*	*7MW2*	*7MW3*	*7MW4*	*7MW5*	*CST1*	*CST2*	*CST3*	*CST4*	*CST5*	*CST6*
QS1		0.002	−0.005	0.024	**0.160**	0.116	−0.108	0.127	0.096	−0.031	**0.261**	−0.103	**0.382**	**−0.211**	**0.172**
QS2	0.002		−0.001	−0.002	−0.029	0.110	0.043	0.045	−0.025	0.083	0.076	0.055	0.018	−0.028	0.064
QS3	−0.005	−0.001		0.004	0.056	0.032	0.000	−0.124	0.022	0.018	−0.020	−0.056	−0.117	−0.043	0.009
QS4	0.024	−0.002	0.004		**0.154**	0.119	−0.008	0.026	−0.014	−0.058	0.065	0.004	**0.296**	0.017	0.149
7MW1	**0.168**	−0.029	0.058	**0.160**		0.016	−0.020	−0.029	0.002	0.151	**0.276**	**0.166**	**0.323**	0.152	**0.324**
7MW2	0.132	0.122	0.035	0.135	0.018		−0.016	0.030	0.045	−0.020	**0.247**	**−0.179**	**0.174**	−0.141	0.144
7MW3	−0.115	0.044	0.000	−0.009	−0.020	−0.015		−0.018	0.011	0.047	−0.112	**0.301**	−0.112	0.082	**0.166**
7MW4	0.135	0.046	−0.129	0.028	−0.030	0.029	−0.018		−0.004	−0.097	0.050	−0.016	0.112	−0.031	−0.115
7MW5	0.106	−0.026	0.024	−0.016	0.002	0.044	0.011	−0.005		0.126	0.148	−0.024	0.078	0.004	**0.178**
CST1	−0.024	0.092	0.018	−0.047	0.130	−0.017	0.048	−0.105	0.124		−0.002	0.008	−0.011	0.010	0.002
CST2	**0.208**	0.088	−0.020	0.054	**0.231**	**0.204**	−0.112	0.053	0.142	−0.002		0.000	0.011	0.013	0.002
CST3	−0.080	0.062	−0.056	0.003	**0.139**	**−0.148**	**0.300**	−0.017	−0.023	0.008	0.000		−0.004	0.016	0.016
CST4	**0.296**	0.020	−0.117	**0.239**	**0.267**	**0.142**	−0.110	0.117	0.074	−0.011	0.011	−0.004		0.009	0.012
CST5	**−0.170**	−0.033	−0.044	0.014	0.131	−0.120	0.085	−0.033	0.004	0.011	0.013	0.017	0.009		0.013
CST6	**0.136**	0.073	0.009	0.123	**0.273**	0.120	**0.166**	−0.122	**0.172**	0.002	0.002	0.016	0.012	0.012	
Results adjusted for Age, Gender, Height, Weight, MMSE and NM															
QS1		0.040	−0.009	−0.038	0.045	0.048	−0.072	**0.168**	0.057	−0.021	0.164	−0.109	**0.268**	**−0.243**	0.158
QS2	0.038		−0.003	0.004	0.007	**0.146**	0.001	0.039	−0.007	0.086	0.100	0.044	0.032	−0.024	0.073
QS3	−0.008	−0.003		−0.007	0.052	0.040	0.007	−0.081	0.009	0.017	−0.009	−0.053	−0.122	−0.049	0.000
QS4	−0.039	0.005	−0.007		0.045	0.052	−0.032	0.037	−0.061	−0.021	0.016	−0.099	0.131	−0.004	0.040
7MW1	0.061	0.010	0.073	0.059		−0.090	−0.123	0.051	−0.065	**0.237**	**0.263**	0.064	**0.210**	0.127	0.174
7MW2	0.056	**0.177**	0.049	0.059	−0.077		0.049	0.012	−0.006	−0.001	**0.184**	−0.164	0.048	−0.154	0.145
7MW3	−0.101	0.002	0.010	−0.044	−0.127	0.059		−0.091	0.101	0.047	0.048	**0.261**	−0.164	0.071	−0.079
7MW4	**0.189**	0.045	−0.095	0.040	0.042	0.012	−0.074		0.037	−0.078	0.103	0.002	0.108	0.002	−0.079
7MW5	0.060	−0.008	0.010	−0.063	−0.051	−0.006	0.077	0.035		0.140	0.080	0.018	0.007	0.010	**0.207**
CST1	−0.016	0.100	0.017	−0.015	**0.155**	−0.001	0.032	−0.078	0.139		−0.012	0.026	0.081	0.005	−0.013
CST2	0.133	0.129	−0.011	0.013	**0.179**	**0.156**	0.035	0.107	0.083	−0.013		0.035	−0.064	0.014	0.018
CST3	−0.086	0.055	−0.059	−0.079	0.044	−0.139	**0.188**	0.002	0.018	0.028	0.036		−0.063	−0.012	−0.116
CST4	**0.249**	0.048	−0.161	0.124	**0.164**	0.047	−0.136	0.129	0.008	0.102	−0.077	−0.073		−0.002	−0.080
CST5	**−0.176**	−0.028	−0.050	−0.003	0.079	−0.119	0.047	0.002	0.009	0.005	0.013	−0.011	−0.002		−0.039
CST6	0.141	0.104	0.000	0.036	0.133	0.138	−0.064	−0.091	**0.241**	−0.016	0.020	−0.13	−0.077	−0.047	
*β* coefficients with a *p*-value < 0.05 are bolded															

ACRONYMS: MMSE: Mini-Mental State Examination; NM: Number of Medications.

**Table 9:** Construct validity analysis: Linear regression between domains and health-related measures.

	IADL	FALL History	CES-D	PA	SPPB	HAND	PWR	TMTA	Gait Speed
QS1	**0.226**	0.231	0.378	−0.088	**−0.721**	−0.966	−4.337	**8.835**	**−0.059**
QS2	−0.022	−0.099	**1.202**	−0.004	−0.127	0.035	−1.732	−4.713	0.006
QS3	0.006	−0.074	−0.129	−0.095	−0.134	−0.402	−1.994	1.431	−0.010
QS4	0.083	−0.230	0.598	−0.097	**−0.577**	**−1.373**	**−7.576**	**9.340**	**−0.039**
7MW1	**0.394**	−0.091	**1.655**	**−0.377**	**−1.317**	**−3.038**	**−19.142**	**14.173**	**−0.205**
7MW2	**0.236**	**0.431**	0.992	**−0.283**	**−0.73**	−0.754	**−7.039**	**8.028**	**−0.092**
7MW3	0.123	−0.079	**1.144**	**−0.175**	−0.071	**−3.851**	**−16.295**	−0.270	−0.011
7MW4	0.044	0.124	−0.379	0.095	−0.037	−0.508	4.473	−1.953	0.021
7MW5	0.068	0.094	0.165	−0.100	**−0.495**	−0.391	−3.496	3.815	**−0.054**
CST1	0.116	0.207	−0.042	0.024	−0.056	−0.334	−2.786	−1.448	**−0.038**
CST2	**0.17**	**0.325**	0.388	−0.097	**−0.914**	0.080	−4.985	**6.339**	**−0.088**
CST3	−0.001	−0.027	**1.479**	−0.108	−0.232	**−2.518**	**−12.943**	−1.101	−0.025
CST4	**0.185**	−0.146	0.129	−0.140	**−0.922**	**−1.550**	−4.761	**10.736**	**−0.070**
CST5	−0.078	−0.064	−0.308	−0.003	**−0.290**	−1.036	**−9.480**	−1.641	−0.016
CST6	**0.170**	0.254	**2.473**	**−0.312**	**−0.728**	**−3.859**	**−22.155**	4.530	**−0.094**
Results adjusted for Age, Gender, Height, Weight, MMSE and NM									
QS1	0.125	0.178	0.071	−0.005	**−0.430**	**−0.764**	−0.855	3.654	−0.024
QS2	0.022	−0.046	**1.266**	−0.019	−0.221	0.232	−2.298	−2.615	−0.006
QS3	0.018	−0.055	−0.265	−0.087	−0.121	−0.213	−0.065	0.893	−0.013
QS4	−0.017	−0.241	0.106	0.017	−0.205	−0.452	−2.679	**4.533**	−0.003
7MW1	**0.189**	−0.328	0.555	**−0.157**	**−0.836**	−0.636	**−6.004**	**6.110**	**−0.161**
7MW2	**0.159**	**0.450**	**1.053**	**−0.263**	**−0.484**	**−1.142**	**−7.652**	2.755	**−0.071**
7MW3	0.064	−0.159	0.011	−0.046	0.107	−0.407	−3.497	−0.892	0.023
7MW4	0.079	0.157	−0.126	0.045	−0.180	**−1.121**	−0.908	0.645	0.006
7MW5	0.023	0.096	0.214	−0.082	**−0.349**	−0.673	−3.001	0.017	**−0.039**
CST1	0.133	0.158	0.063	−0.010	−0.140	−0.196	−0.991	−2.428	**−0.040**
CST2	0.114	**0.389**	0.680	−0.106	**−0.818**	−0.801	**−6.630**	1.341	**−0.072**
CST3	−0.065	−0.028	0.785	0.043	−0.017	−0.774	**−5.934**	−2.237	0.003
CST4	0.058	−0.120	−0.707	0.026	**−0.625**	−0.890	0.845	**5.898**	**−0.037**
CST5	−0.112	−0.087	−0.699	0.054	−0.210	−0.301	**−5.255**	−3.084	−0.002
CST6	0.031	0.178	**1.337**	−0.115	**−0.460**	−0.737	**−6.317**	−1.596	**−0.047**
*β* coefficients with a *p*-value < 0.05 are bolded									

ACRONYMS: CES-D: Center for Epidemiologic Studies Depression Scale; FALL history: declared number of falls; HAND. Hand-Grip strength test; IADL: Instrumental Activities of Daily Living; MMSE: Mini-Mental State Examination; NM: Number of Medications; PA: Physical Activity; PWR. lower extremity muscle power; SPPB: Short Physical Performance Battery; TMTA: Trail Making Test A.

**Table 10:** Linear regression analysis between health-related measures.

	IADL	FALL History	CES-D	PA	SPPB	HAND	PWR	TMTA	Gait Speed
IADL		−0.038	0.714	**−0.295**	**−0.902**	**−1.900**	**−11.782**	**11.805**	**−0.104**
FALL history	−0.014		0.432	−0.050	**−0.217**	−0.618	−2.363	0.222	**−0.022**
CES-D	0.016	0.024		**−0.035**	**−0.049**	**−0.421**	**−2.091**	**1.004**	**−0.009**
PA	**−0.377**	−0.167	**−2.039**		**0.955**	**3.654**	**19.125**	**−9.962**	**0.126**
SPPB	**−0.256**	**−0.160**	**−0.638**	**0.212**		**1.736**	**10.669**	**−7.883**	**0.091**
HAND	**−0.029**	−0.024	**−0.295**	**0.043**	**0.093**		**3.764**	**−1.311**	**0.012**
PWR	**−0.006**	−0.003	**−0.046**	**0.007**	**0.018**	**0.118**		**−0.187**	**0.002**
TMTA	**0.010**	0.000	**0.039**	**−0.006**	**−0.023**	**−0.072**	**−0.328**		**−0.003**
Gait speed	**−2.013**	**−1.083**	**−7.897**	**1.905**	**6.198**	**15.703**	**98.927**	**−76.769**	
Results adjusted for Age, Gender, Height, Weight, MMSE and NM									
IADL		−0.212	−0.313	**−0.133**	**−0.462**	0.056	−1.681	**2.992**	**−0.039**
FALL history	−0.064		0.269	−0.027	**−0.170**	−0.149	0.115	−1.307	−0.010
CES-D	−0.006	0.017		−0.010	0.002	−0.044	−0.321	0.323	−0.003
PA	**−0.177**	−0.121	−0.668		**0.421**	0.398	3.257	−0.577	**0.061**
SPPB	**−0.165**	**−0.202**	0.043	**0.113**		**0.768**	**6.378**	−2.539	**0.070**
HAND	0.002	−0.018	−0.085	0.011	**0.080**		**2.059**	−0.815	**0.007**
PWR	−0.001	0.000	−0.012	0.002	**0.013**	**0.039**		0.050	**0.001**
TMTA	0.003	−0.004	0.017	0.000	−0.007	−0.022	0.072		**−0.001**
Gait speed	**−1.049**	−0.904	−3.734	**1.222**	**5.199**	**5.239**	**52.521**	**−35.582**	
*β* coefficients with a *p*-value < 0.05 are bolded									

ACRONYMS: CES-D: Center for Epidemiologic Studies Depression Scale; FALL history: declared number of falls; HAND. Hand-Grip strength test; IADL: Instrumental Activities of Daily Living; MMSE: Mini-Mental State Examination; NM: Number of Medications; PA: Physical Activity; PWR. lower extremity muscle power; SPPB: Short Physical Performance Battery; TMTA: Trail Making Test A.
